# Aging, cellular senescence and Parkinson's disease

**DOI:** 10.1177/1877718X251316552

**Published:** 2025-02-02

**Authors:** Yue Ma, Madalynn L Erb, Darren J Moore

**Affiliations:** 1 Department of Neurodegenerative Science, Van Andel Institute, Grand Rapids, MI, USA; 2 Aligning Science Across Parkinson's (ASAP) Collaborative Research Network, Chevy Chase, MD, USA

**Keywords:** aging, cellular senescence, neurodegeneration, Parkinson's disease, senolytic

## Abstract

Parkinson's disease (PD) is the most common neurodegenerative movement disorder, affecting 1–2% of people over age 65. The risk of developing PD dramatically increases with advanced age, indicating that aging is likely a driving factor in PD neuropathogenesis. Several age-associated biological changes are also hallmarks of PD neuropathology, including mitochondrial dysfunction, oxidative stress, and neuroinflammation. Accumulation of senescent cells is an important feature of aging that contributes to age-related diseases. How age-related cellular senescence affects brain health and whether this phenomenon contributes to neuropathogenesis in PD is not yet fully understood. In this review, we highlight hallmarks of aging, including mitochondrial dysfunction, loss of proteostasis, genomic instability and telomere attrition in relation to well established PD neuropathological pathways. We then discuss the hallmarks of cellular senescence in the context of neuroscience and review studies that directly examine cellular senescence in PD. Studying senescence in PD presents challenges and holds promise for advancing our understanding of disease mechanisms, which could contribute to the development of effective disease-modifying therapeutics. Targeting senescent cells or modulating the senescence-associated secretory phenotype (SASP) in PD requires a comprehensive understanding of the complex relationship between PD pathogenesis and cellular senescence.

## Introduction

Parkinson's disease (PD) is a neurodegenerative disease that leads to α-synuclein aggregation and degeneration of dopaminergic neurons in the substantia nigra pars compacta (SNpc).^1,2^ The result of this neuropathology is progressively worsening motor symptoms that include resting tremor, rigidity, bradykinesia and poor balance.^1,2^ Often PD also manifests with a myriad of non-motor symptoms that can impact quality of life. Although up to 15% of PD cases have a genetic component, most cases of PD occur sporadically with no known cause.^3^ The risk of developing PD dramatically increases with age suggesting that the aging process creates an environment that promotes, or is less protective against, the development of PD-related neuropathology.^4–6^ Preclinical rodent studies support this hypothesis with older animals exhibiting enhanced susceptibility to PD-linked toxins, α-synuclein pathology or familial PD-linked mutations.^7–10^ Understanding the effects of aging on peripheral tissues and the central nervous system has been a widely studied area of research for many years. Advances in this field will be critical to identifying molecular pathways that contribute to the development and progression of PD neuropathology.

One aspect of organismal aging that may affect the central nervous system is the accumulation of senescent cells. The process of cellular senescence was originally described in tissue culture experiments where diploid cells undergoing multiple rounds of mitosis routinely experienced longer doubling times and eventually ceased dividing with advanced age.^11^ Senescent cells also upregulate and secrete inflammatory cytokines, a phenomenon now known as the Senescence-Associated Secretory Phenotype or “SASP”.^12^ The activation of SASP is thought to promote low level chronic inflammation and has been shown to promote senescence in neighboring cells, which can result in a positive inflammatory feedback loop.^13^

Investigating cellular senescence in the aging brain is a relatively new field of study. Likewise, understanding how cellular senescence in the brain or in peripheral tissues may promote or exacerbate neuropathology is a topic that is actively being explored. For example, like PD, the number one risk factor for developing Alzheimer's disease (AD) is age. Senescent cells accumulate in brain tissue of AD subjects and AD mouse models.^14–17^ Notably, the targeted removal of senescent cells in AD mice has beneficial effects against protein aggregation, neurodegeneration and cognitive decline, offering a promising new therapeutic target.^14,17^ Accordingly, drugs that eliminate senescent cells are currently being evaluated in clinical trials for treating AD patients.^18,19^

Given these promising results, it is surprising how little is known regarding the relationship between senescence and PD neuropathology. This review aims to explore what is currently known about the aging brain and cellular senescence and how senescence relates to known PD neuropathological pathways, while discussing the limited research that is currently available directly examining cellular senescence in PD.

## Aging and Parkinson's disease

Risk for developing PD dramatically increases with advanced age starting at around 55 years.^4–6^ A positive relationship between age and PD neuropathogenesis has also been observed in preclinical rodent models. For example, low doses of rotenone, a PD-related mitochondrial toxin, cause dopaminergic neurodegeneration in the SNpc in aged but not young rats.^8^ Two recent studies show enhanced susceptibility of older rodents to gut injections of α-synuclein preformed fibrils (PFFs). Old or adult rats experience increased pathological α-synuclein in the brain, stomach and enteric nervous system as well as enhanced loss of cholinergic gut neurons compared to young rats 10 weeks after human PFF injections.^7^ In a separate study, 120 days after PFF injection into the duodenum, Challis et al. observed accumulation of pathological α-synuclein in the brainstem of old but not young mice.^9^ Some models of familial PD also show age-dependent neurodegeneration in the SNpc, including D620N *VPS35* knockin mice.^10^ Together these findings support a strong link between aging and susceptibility to PD neuropathogenesis.

Aging is a complex process resulting in a variety of changes that are cumulatively detrimental to longevity and quality of life. These cellular and molecular changes can be grouped into distinct aging hallmarks.^20,21^ The primary hallmarks of aging include genomic instability, telomere attrition, epigenetic alterations, and loss of proteostasis.^20,21^ The antagonistic hallmarks of aging are cellular senescence, deregulated nutrient sensing and mitochondrial dysfunction.^20,21^ The integrative hallmarks of aging are stem cell exhaustion and altered intercellular communication.^20,21^ More recently identified aging hallmarks also include compromised autophagy, microbiome disturbance, altered mechanical properties, splicing dysregulation, and inflammation.^21^

Notably, many of these phenomena are primary features or putative drivers of neuropathology in PD. For example, mitochondrial dysfunction and loss of proteostasis, are both well characterized features of PD in human subjects and are each sufficient to drive degeneration of SNpc dopaminergic neurons in rodents. Likewise, neuroinflammation is a consistent feature of PD neuropathology in human subjects and preclinical rodent models.^22^ Genomic instability, DNA damage and gut microbiome alterations have also recently emerged as features of, and potential drivers of, PD neuropathology.^23,24^ It is currently less clear whether telomere attrition or epigenetic alteration occur in PD.^25^ Cellular senescence is also an area beginning to be explored in postmortem tissue from PD subjects and in cellular and animal models of PD.

### Mitochondrial dysfunction

The relationship between mitochondrial dysfunction and PD has been extensively studied and reviewed elsewhere.^26,27^ Briefly, there is a strong genetic link between mitochondrial dysfunction and PD with loss-of-function mutations in *PARKIN* or *PINK1* causing recessive early-onset familial PD.^28,29^ PINK1 and PARKIN are both critical mitophagy activators and essential for mitochondria quality control.^30–32^ Likewise, treatment with rotenone, which disrupts the function of mitochondrial complex I, causes neurodegeneration of SNpc dopaminergic neurons in mice and rats.^33–36^ Importantly, exposure to rotenone also increases lifetime risk of developing PD in humans.^37^ Histological and biochemical analyses of SNpc tissue from subjects with sporadic PD also show reduced levels of mitochondrial complex I protein or reduced complex I enzymatic activity relative to control tissue.^38–41^ Collectively, mitochondrial dysfunction appears to be a common and important feature of PD neuropathology.

Box 1. Do neurons undergo senescence?

The phenomenon of cellular senescence was originally described through observations of mitotic cells in vitro, as a process through which cells age and stop dividing without dying. Cell cycle arrest was initially considered central to the senescent state. With additional research, it has become clear that the original definition of senescence does not fully apply to all cell types. For instance, post-mitotic cells, which are cells that no longer divide, can become senescent in response to stress.^93^ The effects of cell-cell interactions between different cell types in vivo may also need to be considered when applying this definition. For example, senescent cells can chemo-attract immune cells and are usually cleared by these immune cells, facilitating their exit from anti-apoptotic pathways.^94^

One proposed molecular profile of neuronal senescence is that neurons are metabolically active but with information processing deficits.^95^ Current research investigating neuronal senescence is focused on a multi-marker approach. The markers for these studies are derived from mitotic cellular senescence research, which may not be appropriate for neurons. With aging, neurons display morphological and functional changes, accompanied by changes in proteostasis, redox balance, and Ca^2+^ dynamics; however, it is unclear whether these changes are indicative of senescence.^92^ In recent years, with the development and application of bioinformatics technology and single-cell RNA-sequencing, senescence gene sets have been developed including Senescence Eigengenes and SenMayo.^96,97^ The application of single-cell RNA-sequencing experiments coupled with senescent gene set analysis in brain tissue will further our understanding of senescence in the brain and may lead to the identification of more reliable markers for neuronal senescence.

### Loss of proteostasis

Aggregation of hyperphosphorylated neuronal α-synuclein protein in Lewy bodies and Lewy neurites is a hallmark of PD.^42,43^ Additionally, neuronal aggregation of phosphorylated α-synuclein, driven by delivery of α-synuclein PFFs to the dorsal striatum, produces progressive dopaminergic neurodegeneration in the nigrostriatal pathway.^44^ The presence of aggregated α-synuclein in brain tissue from PD subjects and the ability of α-synuclein aggregates to drive degeneration in the nigrostriatal pathway strongly suggests disrupted proteostasis in PD neuropathology.

Protein degradation is a key component of the proteostasis network. Proteins are degraded by cells through the ubiquitin proteosome system or the autophagy-lysosomal system. Within the autophagy-lysosomal system there are three distinct pathways (1) macroautophagy, (2) chaperone-mediated autophagy (CMA) and (3) microautophagy. Lysosomes play a critical role in each of these pathways and are required for degradation of large misfolded proteins, protein aggregates and damaged organelles. Some of the strongest evidence implicating lysosomal dysfunction in PD neuropathology is the convergence of PD-linked familial mutations and risk variants onto genes that function in the endolysosomal pathway (i.e., *GBA1*, *ATP13A2*, *LRRK2*, *ATP6V0A1*, *SCARB2*, *TMEM175, CTSB*, *GALC).*^45–56^

For example, heterozygous loss-of-function mutations in *GBA1*, which encodes the lysosomal protein β-glucocerebrosidase (GCase), are the most common risk variant for PD.^53–57^ Reduction of GCase protein causes accumulation of lysosomal GluCer and impairs lysosomal protein degradation capacity in neurons.^58^ Additionally, homozygous missense mutations in *ATP13A2*, a lysosomal polyamine export protein, are linked to recessive juvenile-onset familial parkinsonism.^59^ Heterozygous missense mutations have also been found in subjects with young-onset sporadic PD.^45,46^ In dopaminergic cell lines, *ATP13A2* knockdown leads to less acidic lysosomal pH, reduced cathepsin D processing and impaired lysosomal degradative capacity.^60^ *ATP13A2* knockout mice also experience lipofuscin and LAMP1 accumulation throughout the brain, indicative of widespread lysosomal dysfunction.^61^ LRRK2, another familial PD-linked gene, also functions in the endolysosomal pathway via phosphorylation of its Rab substrates.^62^ LRRK2 is recruited to damaged or overloaded lysosomes and appears to promote lysosomal membrane repair through the ESCRT pathway.^63–65^ Notably, expression of the G2019S LRRK2 mutation alters lysosomal morphology and increases lysosomal pH in primary cortical neurons and primary astrocytes.^66,67^

### Genomic instability

Recent studies have also revealed evidence of genomic instability in established rodent models of PD and human PD subjects.^68–71^ DNA damage appears to be a prominent feature of PD neuropathology and may also drive neurodegeneration in the nigrostriatal pathway.^68,70,71^ Histological analysis of SNpc tissue from human PD subjects shows an increase in dopaminergic neurons and microglia with γH2A.X foci, a marker for DNA damage.^68,72^ Additionally, El Saadi et al. recently showed that dopaminergic neurons in the SNpc have relatively high baseline levels of genomic instability in mice.^68^ Low concentration infusion of paraquat into the SNpc also elicits genomic instability in neurons, with dopaminergic neurons exhibiting enhanced sensitivity to this PD-linked toxin.^68^

In addition to producing progressive dopaminergic degeneration in the nigrostriatal pathway as well as motor deficits, striatal injection of α-synuclein PFFs in mice also substantially increases the number of dopaminergic neurons with genomic instability or DNA damage in the SNpc.^68–70^ Likewise, overexpression of human α-synuclein increases the number of dopaminergic neurons in the SNpc experiencing DNA damage.^69^ Treatment of primary neurons with α-synuclein PFFs also induces DNA damage which appears to be driven by accumulation of nitric oxide.^70^ Interestingly, DNA damage can subsequently activate PARP-1, leading to the accumulation of cytosolic poly-ADP-ribose (PAR) polymer.^70^ PAR polymer is increased in both primary neurons and the SNpc in response to α-synuclein PFFs, and in SNpc tissue and CSF from PD subjects.^70,73^ Activation of PARP-1 via DNA damage can lead to a type of controlled cell death called parthanatos, which could be a major contributor to dopaminergic neurodegeneration in PD.^70,73^

Box 2. Is the SASP pro-inflammatory or anti-inflammatory?

The SASP can have different effects depending on cellular context and the microenvironment. Microglia serve as the resident immune cells of the brain. In a healthy state, SASP factors secreted by senescent cells can attract microglia, producing a robust and transient response. This response may facilitate the clearance of senescent cells and maintain a balance of pro-inflammatory and anti-inflammatory responses. However, in the aged brain, the phagocytic capacity of microglia is impaired.^105^ In this situation, SASP factors can lead to prolonged microglia activation without clearance of senescent cells, which will subsequently further promote the release of pro-inflammatory cytokines.^105^ Senescent glia also release pro-inflammatory cytokines, as part of the SASP, contributing to inflammaging, which is a risk factor for neurodegenerative disease.^106,107^

### Telomere attrition

As telomere shortening is a hallmark of aging and can induce cellular senescence, assessing telomere length in PD will be important for understanding the role of aging and senescence in disease onset and progression. So far, analyses of telomere length in PD subjects have been inconsistent.^74^ For example, the ICICLE-PD Study finds that blood cells from PD subjects have shorter telomeres compared to age-matched healthy controls.^75^ However, Asghar et al. finds subjects with PD have longer telomeres in blood and brain tissue.^76^ Notably, several reports have shown telomere dysfunction can occur independent of length.^77^ Longer telomeres signaling a DNA damage response (DDR) have also been implicated during the aging process *in vivo.*^77^ Comprehensive assessment of whether telomere quality is impacting PD neuropathogenesis will require a more detailed analysis of telomere quality in PD subjects. Cellular and animal models of PD could also provide additional insight into the role of telomere quality control in PD neuropathology.

Box 3. Cross talk between DNA damage and PD.

Environmental toxicants are an important factor in the pathogenesis of sporadic PD. Notably, many PD-related toxicants can induce genomic DNA damage and can eventually lead to DNA damage-dependent dopaminergic neurodegeneration.^24^ Whether cellular senescence contributes to this process is worth exploring. Mutations in familial PD-linked genes, including *SNCA*, *LRRK2*, *parkin*, and *DJ-1* can also cause defects in DNA damage repair pathways. Disruption of DNA damage repair pathways may partially explain the accumulation of DNA damage in PD subjects.^24^ Additionally, mitochondrial DNA (mtDNA) damage, can produce reactive oxygen species (ROS), which can further damage genomic DNA, RNA and proteins, potentially contributing to PD neuropathology.^24^

## Cellular senescence

**Figure 1.**
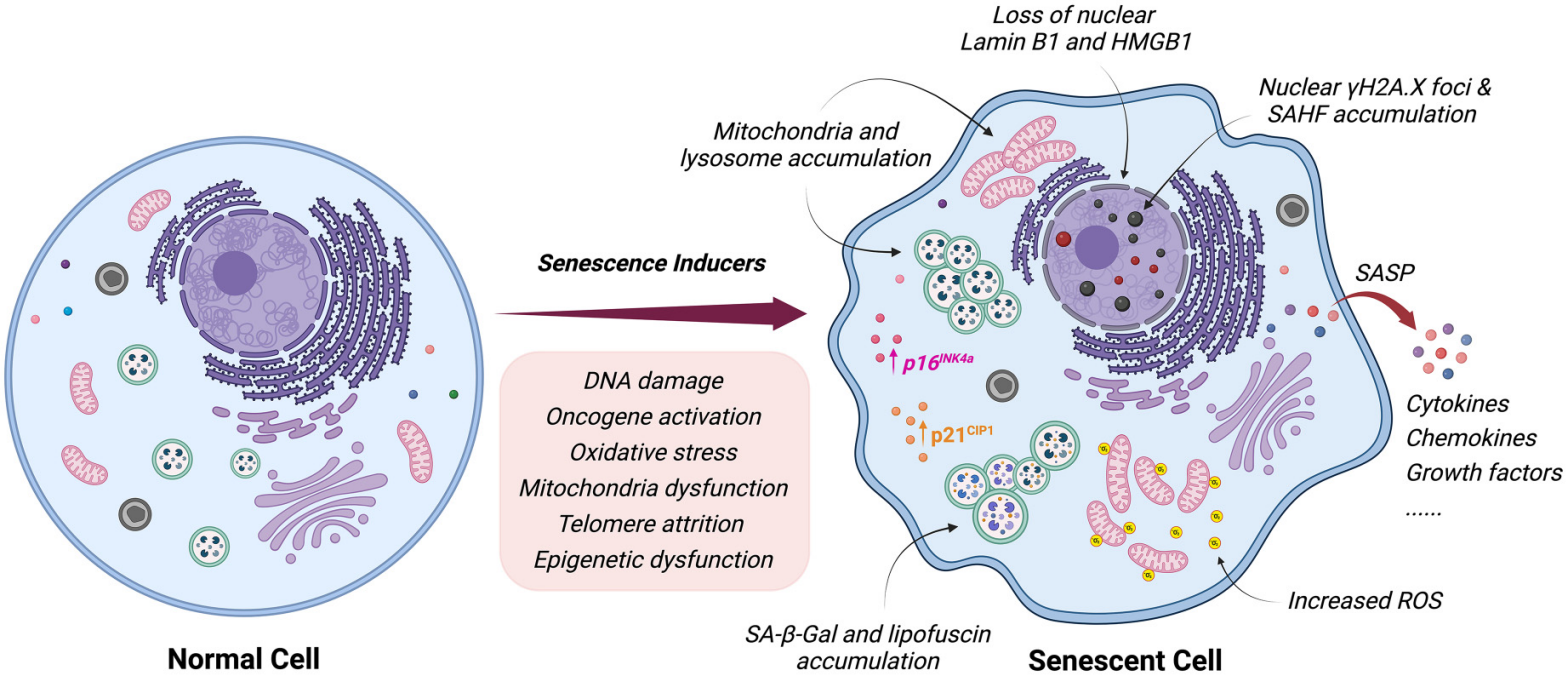
Inducers and hallmarks of cellular senescence. Cells can become senescent in response to a variety of stimuli including DNA damage, oncogene activation, oxidative stress, mitochondrial dysfunction, telomere attrition or epigenetic dysfunction. Senescence can induce many cellular changes including altered morphology, increased senescence-associated β-galactosidase (SA-β-Gal) activity, lipofuscin accumulation, increased expression of p16^INK4a^ and p21^CIP1^, mitochondria and lysosome accumulation, nuclear γH2A.X foci, senescence-associated heterochromatin (SAHF) accumulation, increased reactive oxygen species (ROS), loss of nuclear Lamin B1 and HMGB1 as well as secretion of pro-inflammatory cytokines and chemokines, collectively known as the senescence-associated secretory phenotype (SASP).

The concept of cellular senescence was first proposed by Leonard Hayflick in 1965, based on observations that normal human cells have a limited capacity for replication and eventually enter a state of irreversible growth arrest.^11^ Cellular senescence regulates physiological and homeostatic processes, particularly during embryonic development and wound healing, but can also be a pathological process that contributes to aging, various diseases, and metabolic disorders.^78,79^ Since its discovery, researchers have made substantial progress identifying molecular pathways and markers associated with senescence. Senescent cells exhibit several characteristic features, including altered morphology, increased senescence-associated β-galactosidase activity, and secretion of pro-inflammatory cytokines and chemokines, collectively known as the senescence-associated secretory phenotype (SASP). It is important to note that many senescence-associated molecular and morphological features are present in other cellular states and conditions. The senescent phenotype is also highly heterogeneous and dynamic. Senescence can be induced by a variety of stimuli including DNA damage, activation of oncogenes, oxidative stress, mitochondrial dysfunction, or epigenetic dysfunction.^80,81^ The stimuli and hallmarks of cellular senescence have been summarized in Figure 1 and thoroughly reviewed elsewhere.^82,83^ Here, we discuss the hallmarks of senescence in the context of neuroscience (Box 1), specifically highlighting the relationship between senescence and PD.

### Cyclin-dependent kinase inhibitors (CDKis)

In mitotic cells, senescence occurs when cells enter a permanent state of G_1_ arrest or possibly G_2_.^84,85^ The main drivers of cell cycle arrest in senescent cells are cyclin-dependent kinase inhibitors (CDKis). The gene p16, encoded by *CDKN2A* (*p16^INK4a^*), binds to Cdk4/6 and inhibits phosphorylation of retinoblastoma protein (RB). RB interacts with E2F transcription factors to regulate cell-cycle progression by inhibiting its transcriptional activity.^86^ The resulting downregulation of E2F target genes forces cells to remain in G_1_.^86^ Expression of p16^INK4a^ has been used extensively to detect senescent cells *in vivo* and clearance of p16^INK4a^ -positive senescent cells delays age-related tissue dysfunction.^87–89^ p21 protein encoded by *CDKN1A* (*p21^CIP1^*) inhibits a variety of CDKs, but, paradoxically, is also required for cell cycle progression.^90^ Although p21 is consistently upregulated in response to diverse senescence-inducing stimuli, increases in p21 expression result from a more generic DNA damage repair response.^90^ This upregulation is primarily regulated through direct transactivation via p53, making it difficult to use p21 as a unique senescence marker.^90^ p21 and p16 have been widely used as co-markers to identify senescent cells, but p21^CIP^ and p16^INK4a^ transcripts demonstrate significant heterogeneity across distinct cell types and tissues, frequently exhibiting a lack of co-expression.^91^ Post-mitotic cells, like most adult neurons and glia, are fully differentiated and reside in a terminal G_0_ phase (quiescence). Whether p16 or p21 are informative markers for neuronal or glial senescence is worth pondering (Box 1).^92^

### Secretory phenotype

The SASP is a key characteristic of senescent cells.^98^ It refers to the secretion of various signaling molecules, including cytokines, chemokines, growth factors, and proteases, by senescent cells.^98^ The SASP may also consist of exosomes and ectosomes containing enzymes, microRNA, DNA fragments, chemokines, and other bioactive factors.^98^ The concept of SASP was established by Judith Campisi and her team, who first published on the subject in 2008.^12^ In their study, Coppe et al. described the complex secretory profile exhibited by senescent cells and its implications for aging and cancer.^12^ Since then, the SASP has become a widely recognized phenomenon in the field of cellular senescence and has been extensively studied for its roles in various biological processes.^99^ These secreted factors can have beneficial or detrimental effects (Box 2). For example, the SASP can promote tissue repair, immune surveillance, and clearance of damaged cells. On the other hand, the SASP can contribute to chronic inflammation, tissue dysfunction, and age-related diseases.

Notably, SASP factor upregulation has been observed in brain tissue and primary neuronal cultures. In a long-term in vitro culture of rat cortical neurons, the expression of IL-6 mRNA was significantly higher than in cells cultured for only a few days.^100^ Furthermore, an increased number of IL-6-expressing cortical and Purkinje neurons have been observed in the brains of old mice.^101^ IL-6 is a prominent SASP factor present in neurons and astrocytes, with old astrocytes from a mouse model of AD showing increased levels of IL-6 compared to wild-type (WT) astrocytes.^102^ Increased secretion of IL-6 and the persistent upregulation of IL-1β, IL-8, TNFα, and NOS2 have also been detected in tau-induced senescent astrocytes.^103^ The SASP is highly heterogeneous, with the specific composition of SASP factors varying depending on cell type, senescence-inducing stimulus, and the microenvironment.^104^ Furthermore, different senescent cells within the same tissue or organism can exhibit distinct SASP profiles.^99^ This heterogeneity in SASP factor composition can have important implications for the functional consequences of senescence, as well as for the surrounding tissue and overall organismal health.

### DNA damage response (DDR)

Depending on its magnitude, irreparable DNA damage can induce cellular senescence or apoptosis (Box 3). The DNA damage response (DDR) promotes the recruitment and binding of ATM kinase which drives phosphorylation of H2A.X (γH2A.X) to facilitate the assembly of specific DNA repair complexes.^108,109^ γH2A.X is a potential marker of cellular senescence as persistent γH2A.X may indicate DNA double-strand breaks (DSBs) that remain permanently unrepaired due to cellular senescence.^110^ Furthermore, accumulation of nuclear γH2A.X foci occurs during the pseudo-DNA damage response in senescent cells.^107,111^ It is noteworthy that γH2A.X is widely distributed in the mouse brain, indicating diverse molecular functions in the brain and making it difficult to identify senescent cells using γH2A.X foci alone.^112^ 53BP1, Rad51 and other repair or signaling proteins localized to DSBs or sites of DNA damage-induced replication stress may also be useful indicators of senescence.^113,114^

### Organelle changes

Mitochondria in senescent cells are less functional, showing decreased membrane potential, increased proton leakage, reduced fusion and fission rates, increased mass, and accumulation of tricarboxylic acid (TCA) cycle metabolites.^115^ A recent study from Byrns et al. also shows that loss of select mitochondrial genes in neurons can trigger senescence in neighboring glia.^116^ Interestingly, the presence of senescent glia in the brain may subsequently affect lipid storage in neighboring non-senescent cells.^116^ Increased size and number of lysosomes as well as accumulation of lipofuscin, which are signs of lysosomal dysfunction, are another hallmark of both replicative and stress-induced senescence.^117^ Additionally, the enzymatic activity of the lysosomal senescence-associated beta-galactosidase (SA-β-gal) is a surrogate marker for this enhanced lysosomal content in senescent cells, however the application of this assay is limited. Relatively high SA-β-gal activity has been observed in 3-month-old mice, which given the young age of these mice, is likely unrelated to senescence.^100^ Nuclear loss of lamin B1 or HMGB1 and heterochromatic foci formation are also indicative of cellular senescence.^118,119^ Each of these molecular changes, including increased SA-β-gal activity, can occur in non-senescent cells, which necessitates the use of multiple markers to define cellular senescence.

## Cellular senescence and PD

Recent studies have shown that cellular senescence contributes to the development of neuropathology and progression of neurodegeneration in AD.^120–122^ Accumulation of senescent cells has been observed in brain tissue from AD subjects and in mouse models of AD.^14–17^ Importantly, removal of senescent cells in AD mouse models attenuates Aβ and tau pathology in the brain while also improving cognitive function, implicating senescent cells as pathological contributors to AD.^14,17,123^

Like AD, risk of developing PD dramatically increases with age, suggesting that cellular senescence may contribute to the onset or progression of PD neuropathology.^4–6^ Interestingly, expression levels of the senescence marker p16^INK4a^ and several SASP factors including MMP-3, IL-6, IL-1α, and IL-8 are elevated in SNpc tissue in PD subjects.^124^ GFAP-positive astrocytes in this brain region also show reduced nuclear envelope lamin B1 expression in PD, a hallmark of cellular senescence.^124^ Very few studies have been performed analyzing the expression of senescence markers in brain tissue from PD subjects. As senescence is a complex and heterogenous cell state, comprehensive transcriptomic analyses at the single-cell level will be required to determine whether senescent cells accumulate in different brain regions during disease progression in PD. This type of analysis will also provide critical insight into which cell types are susceptible to senescence during PD neuropathogenesis. Wang et al. recently published a large single-nucleus RNA-sequencing data set collected from SN tissue of PD subjects.^125^ Analyzing these data, and future data sets, for expression of senescence genes using gene enrichment sets such as SenMayo and Senescence Eigengenes will be critical for identifying distinct populations of senescent cells in PD brains and determining their relationship and role in PD neuropathology.^96,97^

Some additional and important tools for evaluating the cellular and molecular mechanisms in PD neuropathology are cellular and animal models. To date, there have been few studies directly assessing senescence in cellular or rodent models of PD. When modeling PD neuropathology there are two main approaches, manipulating the expression of familial PD-linked genes or using PD-related neurotoxins. Examining cellular senescence in diverse models of PD will provide insights into how this cellular state manifests or contributes to the onset and progression of PD neuropathology.

### Genetic models of PD

Aggregation of α-synuclein is a hallmark of PD neuropathology and is associated with neuronal dysfunction and neurodegeneration.^42,43^ Additionally, mutations in *SNCA*, the gene that encodes α-synuclein, or gene duplication or triplication cause familial PD.^126–130^ Accordingly, overexpression of WT *SNCA* or *SNCA* familial PD mutations are well-established strategies to experimentally model PD neuropathology. Interestingly, overexpression of WT *SNCA* in human SH-SY5Y neural cells induces a senescence-like transcriptomic signature including elevated expression of the senescence marker p21.^131^ Overexpression of WT *SNCA* also induces SA-β-gal expression, formation of nuclear γH2A.X foci, and abnormal mitochondrial morphology in these cells, all indicative of a senescent phenotype.^131^ Upregulation of anti-apoptotic pathways, a key feature of cellular senescence, also occurs with WT *SNCA* overexpression in neuronal cells.^132,133^ Yoon et al. also observed a modest increase in mRNA transcript levels of the senescence markers p16 and p53 in motor cortex and hippocampus tissue of young human A53T-*SNCA* transgenic mice.^131^ In another study, viral-mediated overexpression of A53T-α-syn in SNpc for 1 week increased the expression of pro-inflammatory SASP factors, including IL-4, IL-5, and IL-6.^134^ β-gal, p16, p21 and γH2A.X protein levels also increase in the SNpc of the A53T-α-syn overexpressing mice, suggesting that senescence may preceed nigral dopaminergic neurodegeneration in PD.^134^

Human midbrain organoids (hMOs) generated from induced pluripotent stem cells (iPSCs) harboring *SNCA* triplication (3x*SNCA*) may also experience synuclein-related senescence.^135^ Astrocytes and neurons from 3XSNCA hMOs exhibited reduced nuclear lamin B1 compared to control hMOs.^135^ Astrocytes also had increased nuclear γH2A.X foci.^135^ Additionally, expression of *CDKN2A* was upregulated in the 3X*SNCA* hMOs indicating a potentially senescent phenotype.^135^ These results are noteworthy as astrocytes have previously been shown to contribute to the development of α-synuclein-related neuropathology.^136,137^

Microglia also contribute to α-synuclein-related neuropathology and are worth investigating in models of synucleinopathies.^138^ Interestingly, treatment of SH-SY5Y cells with conditioned media from senescent microglia causes an increase in expression of α-syn and induces α-syn aggregation.^139^ A recent study from senescent glial cultures also shows that the capacity for α-synuclein clearance is diminished in senescent glia, likely caused by disruption of the autophagy-lysosomal pathway.^140^ Moreover, in an intrastriatal α-synuclein PFF model, aged microglia exhibited greater accumulation of α-synuclein than younger microglia.^140^ Accumulation of senescent glia in the brain can lead to secretion of inflammatory SASP factors, contributing to chronic inflammation, which can directly affect the health and function of neighboring neurons.^141,142^

Recently, a novel role for LRRK2, a familial PD gene, has been discovered in the DDR pathway with important implications for regulating cellular senescence. LRRK2 increases the expression of p53 and p21 in response to DNA damage in mouse embryonic fibroblasts (MEFs).^143^ G2019S LRRK2, a familial PD mutation, also promotes p53-induced p21 expression in differentiated SH-SY5Y cells and in rat primary neurons.^144^ Additionally, G2019S LRRK2 cells experience increased SA-β-gal activity as well as decreased proteasome and cathepsin D activity, indicative of a senescence-like phenotype.^145^ Dopaminergic neurons in *LRRK2^R1627P^* knockin rats (a PD risk variant) also show some signs of senescence including disrupted Golgi structure, lipofuscin accumulation and dendritic spine degeneration.^146^

Variation at the *Special AT-rich sequence-binding protein 1* (*SATB1*) locus is associated with increased risk for PD.^47^ Interestingly, SATB1 activity is also reduced in PD subjects.^147^ In hESC-derived dopaminergic neurons lacking SATB1, several hallmarks of senescence were observed, including enrichment of senescence pathway genes, increased SA-β-gal activity, increased nuclear diameter, decreased lamin B1 expression, and impaired lysosomal and mitochondrial function.^148^ However, elimination of SATB1 from hESC-derived cortical neurons failed to induce senescence or p21 expression.^148^ These observations suggest that SATB1 activity may be important for repressing p21-dependent senescence specifically in dopaminergic neurons. This finding was further validated in a mouse model of SATB1 reduction, where p21, but not p16, levels were elevated in dopaminergic neurons.^148^ In a follow up study, accumulation of glucocerebrosides was observed in *SATB1* knockout dopaminergic neurons, which contributes to lysosomal and mitochondrial impairment and triggers a cellular senescence-like phenotype.^149^

### Toxicant models of PD

Exposure to the herbicide paraquat is strongly associated with increased lifetime risk for the development of sporadic PD in humans.^150^ Paraquat can also induce progressive dopaminergic neurodegeneration in the nigrostriatal pathway in rodents.^151^ Interestingly, Chinta et al. recently observed induction of senescence markers in cultured human astrocytes in response to paraquat treatment.^124^ These astrocytes displayed several hallmarks of senescence, including increased nuclear size, SA-β-gal activity, increased p16 expression and robust secretion of IL-6, a prominent SASP factor.^124^ A similar senescence phenotype was detected in paraquat-treated mice with increased striatal expression of p16 and IL-6 and decreased nuclear lamin B1 and HMGB1 expression specifically in GFAP-positive astrocytes.^124^ Notably, targeted elimination of p16-positive senescent cells, using the p16-3MR transgenic system, protects paraquat -treated mice from dopaminergic neurodegeneration in the nigrostriatal pathway, demonstrating a pathogenic role for senescent cells in this PD model.^124^

Systemic administration of MPTP is also used to model PD in mice as it causes acute dopaminergic neurodegeneration in the SNpc as well as motor dysfunction.^152^ This model also shows some evidence of cellular senescence, with MPTP-injected mice exhibiting elevated expression of p16, IL-6, and IL-1β in the SNpc.^153^ These gene expression changes may originate from senescent astrocytes as these cells were observed with decreased levels of nuclear lamin B1 expression.^153^ Interestingly, administration of AS-IV, an anti-inflammatory compound, reduces the number of senescent astrocytes in MPTP-injected mice, suggesting a role for paracrine senescence induction through inflammatory SASP factors.^153^

In cells, neurotoxicants can induce senescence in a dose-dependent and time-dependent manner.^154–156^ For example, rotenone treatment in SH-SY5Y cells induces SA-β-gal activity after 24 h.^155,157^ Continuous low-dose rotenone exposure in human trabecular meshwork (HTM) cells, also induces early senescence leading to a late apoptotic signaling cascade.^156,157^ A senescence phenotype was also observed in 6-OHDA-treated SH-SY5Y cells, which could be rescued by treatment with the selective melatonin agonist ramelteon.^158^ MPP + induces senescence in PC12 cells with increased SA-β-gal activity, elevated expression of p53, p21, and p16.^154^ Cultured astrocytes exposed to MPP + also display increased expression of p16 and elevated production of SASP factors, which can be suppressed by AS-IV treatment.^153^ Together, these findings show a consistent induction of senescent phenotypes in response to PD-related mitochondrial toxins in vitro. The evidence for mitochondrial toxin-induced senescence in the brain is also promising but is currently less robust. Additional studies in rodent toxicant models will be required to establish a relationship between senescence and PD-related mitochondrial toxicants.

### Summary of senescence studies in PD

**Figure 2.**
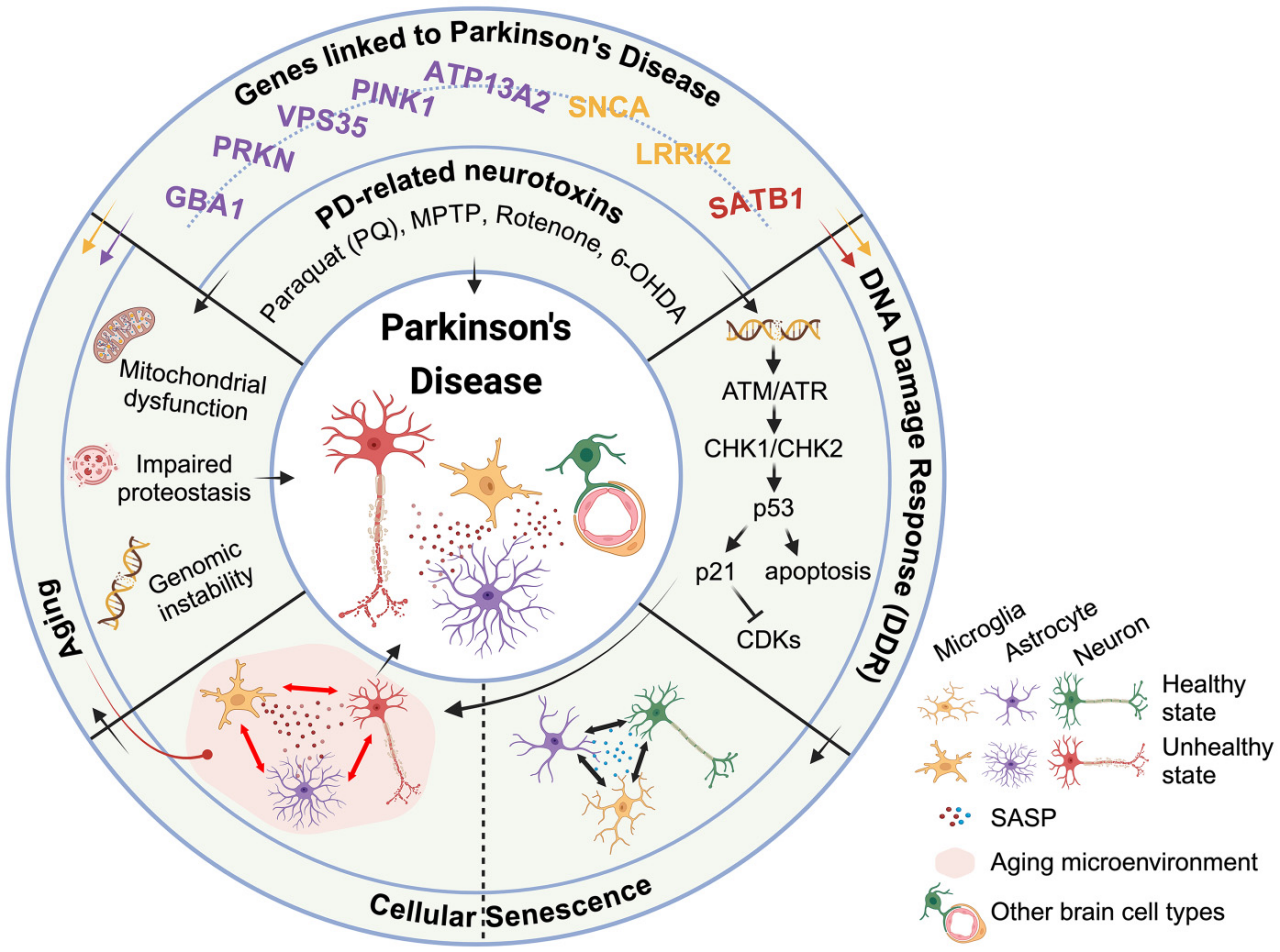
Putative mechanism of the contribution of cellular senescence to neurodegeneration in PD. Environmental toxicants (Paraquat, MPTP, Rotenone) and genes linked to PD (*SNCA*, *LRRK2*, and *SATB1*) can initiate or affect the DNA damage repair (DDR) process (Box 3), leading to cellular senescence. In a healthy state, a small number of senescent cells may persist or be cleared by the immune system. Different types of cells can support each other to maintain homeostasis in the brain. Together with cellular senescence, environmental toxicants (Paraquat, MPTP, Rotenone) and genes linked to PD (*SNCA*, *LRRK2*, *ATP13A2*, *PINK1*, *VPS35*, *PRKN* and *GBA1*) also contribute to the aging process, leading to mitochondrial dysfunction, impaired proteostasis, and genomic instability. With aging, dysfunctional microglia increase the release of pro-inflammatory cytokines and experience reduced phagocytic capacity, which leads to accumulation of senescent cells and SASP factors. The accumulated SASP factors further affect neighboring cells, creating a positive feedback loop that promotes pathological changes or loss of neurons in PD.

Cellular senescence can be induced by genetic perturbations, toxicant exposure or advancing age. According to current research (Table 1), senescence occurs in a variety of cellular and rodent PD models (Box 3). Whether cellular senescence is an initiator or driver of PD neuropathology in these models has not yet been determined. Existing research is still relatively limited, and further studies will be required to assess the complex relationship between PD neuropathogenesis and cellular senescence (Figure 2).

**Table 1.** Cellular senescence studies in PD.

Study	Strategy	Rodent model /*in vitro* model	Senescence feature	Reference
Genetic models	α-synuclein overexpression	SH-SY5Y	Senescence-like transcriptome signature	Yoon et al., 2022^131^
			p21 ↑	
			SA-β-gal ↑	
			H3K9me3 ↑	
			Abnormal mitochondria ↑	
			γH2AX ↑, pATM ↑	
			γH2AX / 53BP1 nuclear foci ↑	
		Tg mice_motor cortex _hippocampus	p16 ↑, p21 ↑ (transcriptional level)	
	α-syn-A53T overexpression	Mice_SNpc	SASP (IL4, IL5, IL6) ↑	Shen et al., 2024^134^
			β-gal ↑, p16 ↑, p21 ↑	
			γ-H2A.X ↑	
			SA-β-gal ↑	
	*SNCA* triplication	Midbrain organoid _astrocyte	Lamin B1 ↓	Muwanigwa et al., 2024^135^
			γ-H2A.X ↑	
			p16 ↑ (transcriptional level)	
	G2019S LRRK2 overexpression	SH-SY5Y (differentiated)	p21 ↑	Ho et al., 2015^144^
		Rat primary neurons	p21 ↑	
		Tg mice_midbrain	p21 ↑	
		SH-SY5Y (differentiated)	β-gal ↑	Ho et al., 2019^145^
	*R1627P LRRK2* knockin	Rat _dopaminergic neurons	lipofuscin ↑	Yang et al., 2024^146^
			Golgi structure disrupted	
			Dendritic spines degenerated	
	*SATB1 *knockout	hESC _dopaminergic neurons	Cellular senescence pathway enriched	Riessland et al., 2019^148^
			p21 ↑	
			SA-β-gal ↑	
			SASP (IGFBP7) ↑	
			Nucleus diameter ↑	
			Lamin B1 ↓	
			lipofuscin ↑	
			Impaired mitochondrial/lysosomal	
	SATB1 knockdown	Mice_midbrain_neurons	p21 ↑	
			SASP ↑	
Toxicant models	Paraquat	Astrocytes	SA-β-gal ↑	Chinta, et al., 2018^124^
			nuclear size ↑	
			p16 ↑	
			SASP (IL6) ↑	
		Mice_striatum_astrocytes	p16 ↑	
			SASP (IL6) ↑	
			Lamin B1 ↓	
	MPTP/MPP+	Mice_SNc_astrocytes	p16 ↑	Xia et al., 2020^153^
			SASP (IL6, IL1β) ↑	
			Lamin B1 ↓	
		Astrocytes	p16 ↑	
			SASP ↑	
		PC12	SA-β-gal ↑	Li et al., 2021^154^
			p16 ↑, p21 ↑	
	Rotenone	SH-SY5Y	SA-β-gal ↑	Yu et al., 2013^155^
		SH-SY5Y (differentiated)	β-gal ↑, p21 ↑	Ho et al., 2021^157^
	6-OHDA	SH-SY5Y	SA-β-gal ↑	Liu et al., 2021^158^
			p21 ↑	
PD subjects	N/A	N/A	p16 ↑	Chinta et al., 2018^124^
			SASP (MMP-3, IL6, IL1α, IL8) ↑	
			Lamin B1 ↓	

## Therapeutic approaches targeting senescent cells

Since the accumulation of senescent cells can damage surrounding tissue through secretion of inflammatory SASP factors, compounds that facilitate removal of senescent cells or inhibit the SASP can have therapeutic benefits to extend lifespan and healthspan.^159^ Many senescent cells, including those that exhibit SASP, upregulate anti-apoptotic pathways (Senescent Cell Anti-apoptotic Pathways or SCAPs). Senolytic compounds inhibit various aspects of SCAPs to promote apoptosis of senescent cells, with minimal effects on healthy non-senescent cells.^160^ Well characterized senolytic compounds include Dasatinib, Quercetin, Fisetin, and Navitoclax.^160^ Dasatinib is a tyrosine receptor kinase inhibitor with multiple targets including ABL, SRC family kinases, platelet derived growth factor receptors (PDGFR), tyrosine receptor kinases and ephrin receptors.^161^ The senolytic properties of Dasatinib likely result from inhibition of EFNB-1/3 signaling.^162^ Quercetin and Fisetin are flavonoids that each inhibit phosphatidylinositol 3-kinases (PI3Ks) with Quercetin also inhibiting NFκB signaling while Fisetin inhibits the PI3K/AKT/mTOR pathway.^162–164^ Navitoclax is a Bcl-2 family inhibitor.^165,166^ The heterogeneity of senescent cells makes it difficult to target this population with individual compounds. Combining senolytic compounds to target diverse SCAPs improves the efficiency of this approach. For example, combined use of Dasatinib and Quercetin (D + Q) effectively reduces senescent cells in diverse mouse models of age-related disorders.^159,160,162^

D + Q treatment may also be an effective therapeutic to prevent AD neuropathology and cognitive decline. Accumulation of senescent cells has been observed in brain tissue of rodent AD models and human AD subjects.^14–17^ Interestingly, oral administration of D + Q effectively reduces the number of senescent oligodendrocyte precursor cells (OPCs), and prevents cognitive decline, in aged APP/PS1 mutant mice.^14^ D and Q were also observed in brain tissue following oral administration in mice, demonstrating the ability of these compounds to cross the blood brain barrier, and suggesting a direct effect on senescent OPCs.^14^ Senescent gene expression profiles are also associated with neurofibrillary tangles (NFTs) in human AD subjects and in rTg(tauP301L)4510 (Tau_NFT_) mutant mice.^17^ Similar to the APP/PS1 mutant mice, D + Q treatment in Tau_NFT_ mice attenuates neuronal atrophy, reduces the number of NFT-bearing neurons and reduces the expression of inflammatory SASP factors.^17^

As D + Q is generally well-tolerated in humans, this senolytic cocktail may be a promising approach for treating AD.^167^ Accordingly, an open-label pilot study testing the safety of D + Q treatment, as well as blood-brain barrier penetration, in a small number of older subjects with early AD has recently been completed.^18,19^ Gonzales et al. report well tolerated D + Q treatment in 5 AD subjects over 12 weeks.^18,19^ D and Q were increased in the blood plasma of all 5 subjects and D was detected in the CSF of 4 subjects.^18,19^ This study is an exciting step towards assessing whether treatment with senolytic compounds will effectively attenuate neuropathology or neurodegeneration in AD subjects. Determining the effects of D + Q on cognitive function and neuropathology will require larger studies conducted over longer time periods.

Whether targeting senescent cells for removal offers a promising treatment opportunity for PD subjects remains unknown. As there is growing evidence that tau plays an important role in PD neuropathology, the therapeutic effects of D + Q in Tau_NFT_ mice may also be relevant for treating PD. Genetic variation at the *MAPT* locus, which encodes tau, affect risk for developing PD and risk for developing cognitive decline or PD dementia in PD subjects.^168–171^ Additionally, tau aggregation is often found along-side Lewy body pathology in PD subjects.^172–174^ Together, these observations implicate tau as a likely contributing factor in PD neuropathology. As clinical trials evaluating the effects of D + Q treatment in AD subjects progress, it will be important to consider whether this may be an effective treatment for PD subjects. Identifying PD subjects experiencing high levels of tau pathology could also be an important factor in predicting the effectiveness of D + Q treatment. Currently, the contribution of cellular senescence in the initiation or progression of PD has not been established. Additional studies examining senescent gene expression profiles in tissues from PD subjects and in rodent models will be critical for understanding the relationship between senescence and PD neuropathology. If a positive relationship is established, further insight into the molecular profile of senescent cells that contribute to PD neuropathology could, hypothetically, provide insights into which senolytic compounds may be most therapeutic. Preclinical experiments assessing the effects of these senolytic compounds in well-established rodent models of PD would also need to be performed prior to initiating clinical trials.

## Challenges and future perspectives

Senescent cells are heterogeneous and have diverse effects on surrounding tissue depending on cell type and microenvironment. There is no unique or specific marker for detecting senescence, and especially no way to detect increased senescence in PD subjects. Developing reliable biomarkers is a critical step and will be necessary to reliably detect and characterize senescent cells in the brain (Box 1). The use of ‘omics techniques to quantify various macromolecules, possibly at the single cell level to include intrapopulation variability, will be a preferred avenue for studying senescence, particularly for characterizing senescent cells in tissues. Senescent cells can contribute to neuroinflammation through the SASP, exacerbating neuronal dysfunction and degeneration. Disentangling the complex interplay between senescence, inflammation, and neurodegeneration in PD will require detailed mechanistic studies (Box 2). Targeting senescent cells or modulating the SASP for therapeutic purposes in PD requires a more comprehensive understanding of their specific contributions to disease pathogenesis. The lack of specific senescence markers greatly hinders testing of therapies counteracting senescence in PD. Identifying strategies to selectively eliminate senescent cells without affecting healthy cells will also be a major goal. Meanwhile, widespread removal of senescent cells in the brain could affect immune surveillance and disrupt tissue repair. Balancing senescence reduction with tissue repair requirements will be critical for utilizing senolytics to effectively treat neurodegenerative diseases. Although studying senescence in PD presents challenges, it also holds promise for advancing our understanding of disease mechanisms and developing effective disease-modifying treatments. Collaborative efforts combining basic research, clinical studies, and therapeutic development are essential to address these challenges and realize the potential of senescence-targeted approaches for PD.
